# A two-stage strategy to facilitate atypical minor duodenal papilla cannulation in pediatric patients: a case series

**DOI:** 10.1055/a-2599-9723

**Published:** 2025-07-01

**Authors:** Sheng Ding, Biao Gong, Zhaohui Deng, Tianao Zhang

**Affiliations:** 1 426116 Department of Gastroenterology, Shanghai Childrenʼs Medical Center Affiliated to Shanghai Jiaotong University School of Medicine, Shanghai, China; 2 671777 Department of Gastroenterology, Guizhou Branch of Shanghai Childrenʼs Medical Center Affiliated to Shanghai Jiaotong University School of Medicine, Guiyang, China; 3 66329 Gastroenterology, Shanghai Shuguang Hospital Affiliated to Shanghai University of Traditional Chinese Medicine, Shanghai, China

A 5-year-old girl diagnosed with chronic pancreatitis, who had previously undergone a failed ERCP. One month later, she was readmitted for a repeat ERCP at our center (case 1). Pancreatography revealed incomplete pancreas divisum with concomitant chronic pancreatitis ( Fig. 1 ). Direct cannulation attempts at both the major and minor papillae were unsuccessful. During another attempt at the major papilla, the hydrophilic tip of the guidewire (0.035 inch, Innovax Medical) serendipitously advanced into the minor papilla. After careful evaluation, the “ventral-to-dorsal pancreatic duct” pathway was retained, and the pathway was enlarged with a COOK 6Fr dilation catheter. A 5 Fr-5 cm pancreatic stent (COOK) was then implanted, with its tip located outside the minor papilla and its pigtail segment positioned at the major papilla. The girl developed mild post-ERCP pancreatitis (PEP). Six weeks later, a second-stage ERCP was performed. Under guidance from the stent, a guidewire was inserted through the minor papilla using a sphincterotome, followed by the removal of the pancreatic stent with forceps. The dorsal duct was further dilated using a 7 Fr catheter, and a 7 Fr-7 cm stent was successfully placed. The patient experienced no complications following the second ERCP ( Video 1 ).

**Fig. 1:**
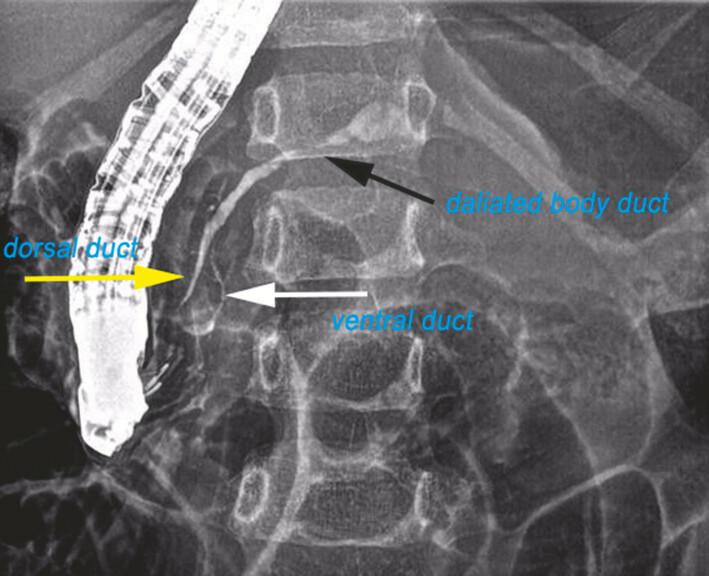
Incomplete pancreas divisum imaging. Hypoplastic ventral pancreatic duct (white arrow), dominant dorsal duct (yellow arrow), and dilated body segment of pancreatic duct (black arrow).

A two-stage strategy to facilitate atypical minor duodenal papilla cannulation in pediatric patients.Video 1

By implementing this two-stage approach, we successfully replicated the protocols in two subsequent cases (cases 2 and 3). Case 2 developed mild PEP during the first-stage ERCP, and Case 3 manifested hyperamylasemia following each stage. All of the complications demonstrated rapid resolution with conservative management.

Compared to conventional rendezvous techniques, the two-stage ERCP strategy in pediatric populations offers three key advantages:

1. Avoidance of papillary trauma: This strategy eliminates shearing injury caused by guidewire traction at the minor papilla, thereby preserving its integrity and minimizing pancreatic duct injury 1 .
2. Risk mitigation in anatomically challenging cases: It avoids high-risk sphincterotomy of small-based minor papillae within narrow intestinal lumens, particularly due to childrenʼs anatomical constraints 2 .
3. Operational efficiency: The technique reduces the need for additional instruments (needle knives, endoscopic ultrasonography, etc.) and shortens operative duration, thereby enhancing procedural safety and reducing costs 3 4 .

Endoscopy_UCTN_Code_TTT_1AR_2AB

## Notes

Endoscopy E-Videos https://eref.thieme.de/e-videos
*E-Videos* is an open access online section of the journal *Endoscopy* , reporting on interesting cases and new techniques in gastroenterological endoscopy. All papers include a high-quality video and are published with a Creative Commons CC-BY license. Endoscopy E-Videos qualify for HINARI discounts and waivers and eligibility is automatically checked during the submission process. We grant 100% waivers to articles whose corresponding authors are based in Group A countries and 50% waivers to those who are based in Group B countries as classified by Research4Life (see: https://www.research4life.org/access/eligibility/ ). This section has its own submission website at https://mc.manuscriptcentral.com/e-videos .
